# A New 9,10-Dihydrophenanthrene and Cell Proliferative 3,4-δ-Dehydrotocopherols from *Stemona tuberosa*

**DOI:** 10.3390/molecules20045965

**Published:** 2015-04-03

**Authors:** Yun-Seo Kil, Jiyoung Park, Ah-Reum Han, Hyun Ae Woo, Eun-Kyoung Seo

**Affiliations:** College of Pharmacy, Graduate School of Pharmaceutical Sciences, Ewha Womans University, Seoul 120-750, Korea; E-Mails: k_yunseo@naver.com (Y.-S.K.); 37301012@hanmail.net (J.P.); arhan@ewha.ac.kr (A.-R.H.)

**Keywords:** *Stemona tuberosa*, Stemonaceae, 9,10-dihydrophenanthrene, 3,4-δ-dehydrotocopherol, chiral separation, cell proliferation

## Abstract

A new compound, 9,10-dihydro-5-methoxy-8-methyl-2,7-phenanthrenediol (**1**), was isolated from the roots of *Stemona tuberosa* Lour. (Stemonaceae) together with two new optically active compounds, (2*S*,4'*R*,8'*R*)-3,4-δ-dehydrotocopherol (**2**) and (2*R*,4'*R*,8'*R*)-3,4-δ-dehydrotocopherol (**3**). The structures of compounds **1**–**3** were determined on the basis of spectroscopic data analysis. Compounds **2** and **3** were each purified from a stereoisomeric mixture of **2** and **3** by preparative HPLC using a chiral column for the first time. The absolute configurations at C-2 of **2** and **3** were determined by Circular Dichroism (CD) experiments. As a part of the research to find natural wound healing agents, all isolates and the mixture of **2** and **3** were evaluated for their cell proliferative effects using a mouse fibroblast NIH3T3 and a HeLa human cervical cancer cell line. As a result, **1**, **2**, **3**, or the mixture of **2** and **3** showed 41.6%, 78.4%, 118.6%, 38.2% increases of cell proliferation in the mouse fibroblast NIH3T3 respectively, compared to 28.4% increase of δ-tocopherol. Moreover, none of them induced cancer cell proliferation. Therefore, 3,4-δ-dehydrotocopherols, especially pure isomers **2** and **3** can be suggested as potential wound healing agents.

## 1. Introduction

The dried root of *Stemona tuberosa* Lour. (Stemonaceae), known as Stemonae Radix, is called “back-bu-geun” in Korea. This herbal medicine was traditionally used as an antitussive and insecticidal drug in Asia [1]. Previous phytochemical reports on *S. tuberosa* mention alkaloids [2,3,4,5], stilbenoids [6,7], phenanthrene [7], and some minor dehydrotocopherol derivatives as isomer mixtures containing δ-tocopherol [8]. The alkaloids and stilbenoids were reported as the antitussive [2,3,4] and antibacterial agents [7] while the dehydrotocopherol derivatives exhibited antioxidant activities [8].

In the general wound healing processes, fibroblasts play important roles in the replacement of damaged cells by synthesizing collagen proteins [9], so natural compounds found to induce cell proliferation in fibroblasts can be suggested as potential wound healing agent candidates.

**Figure 1:**
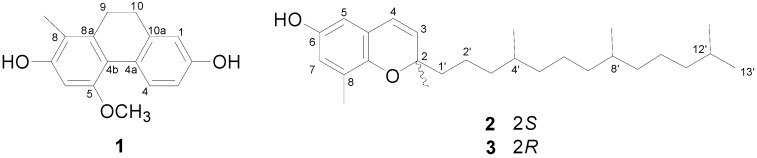
Chemical structures of the isolates **1**–**3** from the root of *S. tuberosa*.

In the present study, a new compound, 9,10-dihydro-5-methoxy-8-methyl-2,7-phenanthrenediol (**1**) was isolated from *S. tuberosa*, along with (2*S*,4'*R*,8'*R*)-3,4-δ-dehydrotocopherol (**2**) and (2*R*,4'*R*,8'*R*)-3,4-δ-dehydrotocopherol (**3**) (Figure 1). Preparative HPLC using a chiral column was performed for the purification of compounds **2** and **3** from their stereoisomeric mixture. All isolates and the mixture of **2** and **3** were tested for their cell proliferation activities on NIH3T3 and HeLa cell lines. Herein, we describe the isolation and structure elucidation of **1**–**3** and suggest potent wound healing agent candidates.

## 2. Results and Discussion

### 2.1. Structure Elucidation of Compound ***1***

**Figure 2:**
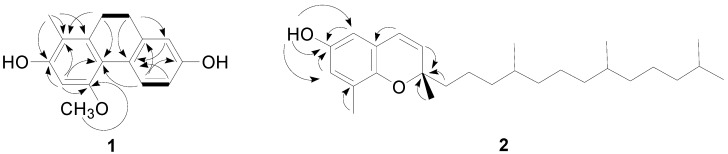
Key COSY (▬) and HMBC (→) correlations of **1** and **2**.

Compound **1** was isolated as a light brown oil and its molecular formula was assigned as C_16_H_16_O_3_ based on a protonated molecular ion peak at *m/z* 257.1172 [M+H]^+^ (calcd. for C_16_H_17_O_3_, 257.1172) in HRESIMS. The ^1^H- and ^13^C-NMR spectra of **1** exhibited four aromatic protons at δ_H_ 7.95 (d, *J* = 8.6 Hz), 6.62 (d, *J* = 2.4 Hz), 6.59 (dd, *J* = 8.6, 2.4 Hz), and 6.43 (s) with 12 aromatic carbons, indicating the presence of two aromatic rings in **1**. Additionally, two methine groups appeared at δ_H_ 2.66 (2H, m, H_2_-9)/δ_C_ 27.5 and 2.60 (2H, m, H_2_-10)/31.1, which showed the COSY correlation between H_2_-9 and H_2_-10 and the HMBC cross peaks of H_2_-9 with C-8/C-4b and H_2_-10 with C-1/C-4a. With another important HMBC correlation of H-4 with C-4a, the above observations provided evidence of **1** being a 9,10-dihydrophenanthrene derivative [7,10]. The residual signals at δ_H_ 3.78 (3H, s) and 2.11 (3H, s) were assignable to a methoxy group at C-5 and a methyl group at C-8 and the positions of these functionalities were confirmed by the HMBC correlations of OCH_3_-5 with C-5 and CH_3_-8 with C-7/C-8/C-8a and NOESY cross peaks of H-1/H_2_-10, OCH_3_-5/H-4 and H-6, and CH_3_-8/H_2_-9 (Figure 2). The carbon resonances of two quaternary carbons at δ_C_ 156.1 (C-2) and 155.4 (C-7) were indicative of the presence of hydroxy groups at C-2 and C-7. The unambiguous assignments of ^1^H- and ^13^C-NMR resonances were conducted by further interpretations of the COSY, NOESY, HSQC, and HMBC data and by comparison of those with the literature values of the known compound, racemosol [7]. The only difference of **1** from the reported compound was the absence of a methoxy group at C-1. Therefore, the structure of **1** was elucidated a new compound, 9,10-dihydro-5-methoxy-8-methyl-2,7-phenanthrenediol.

### 2.2. Chiral Separation and Structure Determination of Compounds ***2*** and ***3***

**Figure 3:**
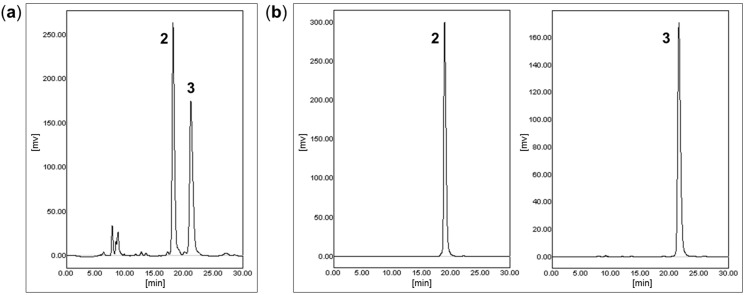
(**a**) HPLC chromatograms of mixture of **2** and **3**; (**b**) HPLC chromatograms of pure isomers **2** and **3** [column: ChiralPak IA (5 μm, 250 × 10 mm); mobile phase: *n*-hexane–EtOH, 99:1, *v/v*; flow rate: 3 mL/min; detection: UV 265 nm; *t*_R_: (**a**) **2** 18.1, **3** 21.1 min; (**b**) **2** 18.8, **3** 21.5 min].

A subfraction was obtained by repeated column chromatography from the EtOAc extract of *S. tuberosa*. This subfraction appeared as a single peak in normal phase HPLC as described in the Experimental Section. However, the corresponding ^13^C-NMR spectrum showed overlapped peaks, which suggested that the subfraction was a mixture of isomers. This mixture was applied to a chiral column (ChiralPak IA, Daicel, Osaka, Japan, 5 μm, 250 × 10 mm), and effectively separated by preparative HPLC to afford two pure stereoisomers, **2** (*t*_R_ 18.1 min, 15 mg) and **3** (*t*_R_ 21.1 min, 15 mg) as shown in Figure 3.

Compounds **2** and **3** showed protonated HRESIMS molecular ion peaks at *m/z* 401.3412 and 401.3416, respectively, corresponding to the same elemental formula of C_27_H_44_O_2_. The ^1^H- and ^13^C-NMR data of both compounds were very similar to the reported values of 3,4-δ-dehydrotocopherol [8]. The planar structures of **2** and **3** were confirmed to correspond to 3,4-δ-dehydrotocopherol by the detailed analysis of their HMBC and NOESY data (Figure 2). However, the ^1^H-, ^13^C-NMR data of **3** were slightly different from those of **2**. The ^1^H-NMR spectrum of **3** was comparable with that of **2**, except for the aliphatic protons of C-2' appearing at δ_H_ 1.41 (2H, m) in **3** instead of δ_H_ 1.46 and 1.36 (each 1H, m) in **2**. Two methyl groups, CH_3_-4' and CH_3_-8' resonated at δ_H_ 0.834 (6H, d, *J* = 6.4 Hz) in **3** instead of δ_H_ 0.841 (3H, d, *J* = 6.4 Hz) and 0.830 (3H, d, *J* = 6.6 Hz) in **2**. Moreover, the carbon signals of C-1' and CH_3_-4' in **3** appeared at δ_C_ 40.97 and 19.6 respectively, whereas they were observed at δ_C_ 41.01 and 19.7 in **2**.

**Figure 4:**
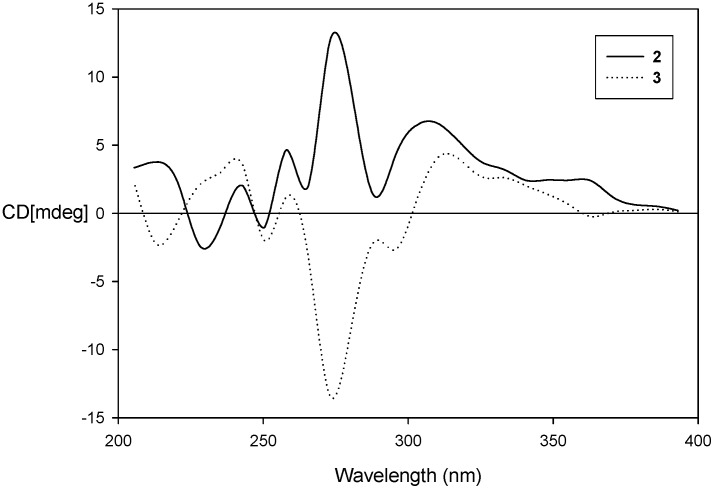
CD data of compounds **2** and **3**.

To solve the absolute configurations of **2** and **3**, Circular dichroism (CD) experiments were performed. The Cotton effect of the 260–270 nm transition has been used to determine the absolute configurations of chiral centers near a styrene chromophore, and subgroups on the chromophore do not affect the sign of the Cotton effect [11]. The CD curve of **2** displayed a positive Cotton effect at 278 nm (as shown in Figure 4), thus the absolute configuration of C-2 was determined to be *S* [12]. Conversely, a negative Cotton effect at 275 nm was shown in the CD data of **3** and the absolute configuration of C-2 was confirmed as *R*. As a result, the structures were assigned as (2*S*,4′*R*,8′*R*)-3,4-δ-dehydrotocopherol (**2**) and (2*R*,4′*R*,8′*R*)-3,4-δ-dehydrotocopherol (**3**), respectively. This is the first report on the separation of **2** and **3** as optically active compounds, together with determination of the absolute configurations for both isomers.

### 2.3. Cell Proliferative Effects

**Figure 5:**
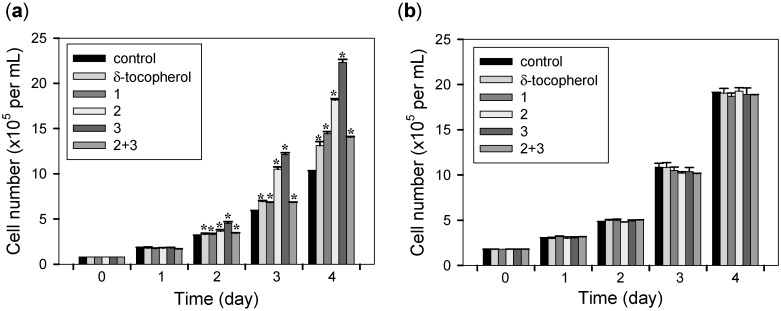
Cell proliferation activities of δ-tocopherol, all isolates, or the mixture of **2** and **3** (**a**) on NIH3T3 cells and (**b**) on HeLa cells. Equal numbers of cells were treated with DMSO alone (control), 10 µM of δ-tocopherol, all isolates, or the mixture of **2** and **3** (**2** + **3**) and cells were counted every 24 h. Data represent the mean ± s.d. of three independent experiments. * *p* < 0.05 *vs.* control (DMSO alone).

The cell proliferation effects of all isolates and the mixture of **2** and **3** were evaluated on mouse fibroblasts NIH3T3 and human cervical cancer cells HeLa (Figure 5). In terms of chemical relevance with the tested compounds, δ-tocopherol was used for comparison in the present biological study. Cells were treated with 10 µM of all isolates, the mixture of **2** and **3**, δ-tocopherol, or DMSO alone and cell counts were performed every 24 h. Daily microscopic examination of the cells showed no differences in cell attachment or morphology between compound-treated and control cells during the incubation times. As a result, all isolates or the mixture of **2** and **3** increased the number of NIH3T3 cells (41.6%, 78.4%, 118.6%, or 38.2% respectively) compared to the DMSO-treated control cells after 4 days incubation with compounds. Incubation with δ-tocopherol also led to a significant but smaller increase of NIH3T3 cells (28.4%). Interestingly, none of these compounds had a proliferative effect on HeLa cells, indicating their ideal wound healing possibility.

**Figure 6:**
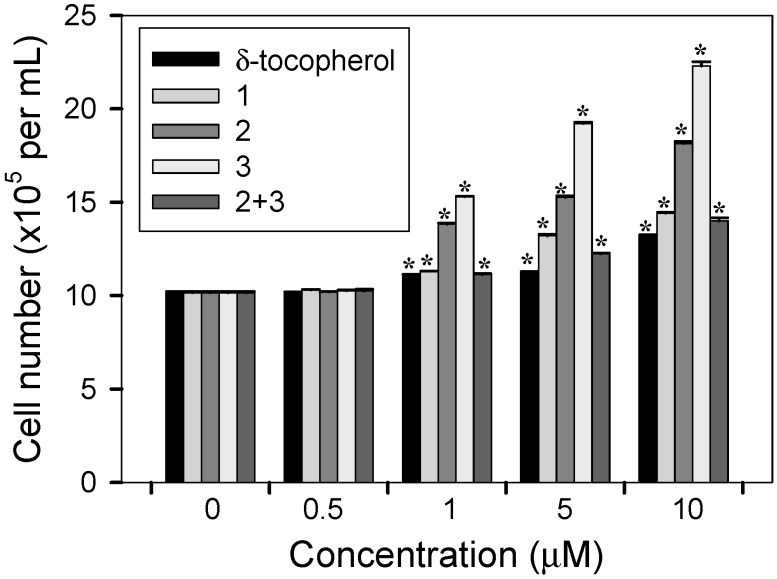
Dose-dependency of cell proliferation activities of δ-tocopherol, all isolates, or the mixture of **2** and **3** on NIH3T3 cells. Equal numbers of cells were treated with DMSO alone (control), indicated concentrations of δ-tocopherol, all isolates, or the mixture of **2** and **3** (**2** + **3**) and cells growth was determined after 4 days by counting cell numbers. Data represent the mean ± s.d. of three independent experiments. * *p* < 0.05 *vs.* control (0 µM).

The compounds promoted fibroblast growth dose-dependently. NIH3T3 cells were treated with the indicated concentrations of δ-tocopherol, all isolates, and the mixture of **2** and **3**. After 4 days of incubation, cell numbers were counted. Growth promotion rates in NIH3T3 cells by 0.5, 1, 5, and 10 µM concentrations of compound **3** were 1.0%, 50.6%, 89.5%, and 119.6%, respectively (* *p* < 0.01 *vs.* control) (Figure 6). 

Similar dose-dependent growth promotion by compound **2** was also observed. Growth promotion rates in NIH3T3 cells by 0.5, 1, 5, and 10 µM concentrations of compound **2** were 0.5%, 35.7%, 49.6%, and 78.7%, respectively (* *p* < 0.01 *vs.* control) Incubation with compounds **1**, the mixture of **2** and **3**, and δ-tocopherol also led to a significant but smaller dose-dependent growth promotion effect.

## 3. Experimental Section

### 3.1. General Procedures

δ-Tocopherol was purchased from Sigma (St. Louis, MO, USA). Optical rotations were measured on a P-1010 polarimeter (Jasco, Tokyo, Japan). UV spectra were recorded on a U-3000 spectrophotometer (Hitachi, Tokyo, Japan). CD spectra were obtained using a J-810 CD-ORD spectropolarimeter (Jasco). HR-ESI mass spectrometric analyses were performed with a Waters ACQUITY UPLC system (Waters, Milford, MA, USA) coupled to a Micromass Q-Tof Micro mass spectrometer and Agilent 6220 Accurate-Mass TOF LC/MS system (Agilent, Santa Clara, CA, USA). The 1D and 2D NMR experiments were performed on a Unity Inova 400 MHz FT-NMR instrument (Varian, Palo Alto, CA, USA) with tetramethylsilane (TMS) as an internal standard. Thin-layer chromatographic (TLC) analysis was performed on Kieselgel 60 F_254_ (Merck, Darmstadt, Germany), with visualization under UV light (254 and 365 nm) and 10% (*v/v*) sulfuric acid spray followed by heating (120 °C, 5 min). Silica gel (230–400 mesh, Merck) and Sephadex LH-20 (GE Healthcare, Uppsala, Sweden) were used for column chromatography (CC). Preparative HPLC was carried out on an Acme 9000 system (Young Lin, Anyang, Korea) equipped with CHEMOSORB 5Si (5 μm, 250 × 20 mm, Chemco, Osaka, Japan) and ChiralPak IA (5 μm, 250 × 10 mm, Daicel, Osaka, Japan).

### 3.2. Plant Material

The dried roots of *S. tuberosa* were purchased from the Insan Oriental Herbal Market in Seoul, Korea. The sample was identified by Prof. Je-Hyun Lee (College of Oriental Medicine, Dongguk University, Gyeongju, Korea). A voucher specimen (No. EA322) has been deposited at the Natural Product Chemistry Laboratory, College of Pharmacy, Ewha Womans University (Seoul, Korea).

### 3.3. Extraction and Isolation

The roots of *S. tuberosa* (10 kg) were extracted with MeOH (3 × 15 L) overnight at room temperature. The solvent was evaporated *in vacuo* to afford a MeOH extract (3.6 kg), which was then suspended in distilled water, and successively partitioned with hexanes (3 × 5 L), EtOAc (6 × 5 L), and *n*-BuOH (6 × 5 L). The EtOAc extract (98 g) was subjected to silica gel column chromatography (CC) (CH_2_Cl_2_–MeOH, 100:0 to 0:100, *v/v*) to obtain 12 fractions (F01–F12). F03 (0.5 g), eluted with CH_2_Cl_2_–MeOH (999:1) from the first separation, was subjected to Sephadex LH-20 CC (100% MeOH) to give six subfractions (F03.01–F03.06). Subfraction F03.03 (176 mg) was separated by preparative HPLC (CHEMOSORB 5Si, *n*-hexane–EtOAc, 9:1, *v/v*, 2 mL/min, UV 265, 330 nm) to yield an isomer mixture (*t*_R_ 59.4 min, 92 mg). The isomer mixture was purified by chiral preparative HPLC (ChiralPak IA, *n*-hexane–EtOH, 99:1, *v/v*, 3 mL/min, UV 265 nm) to afford compounds **2** (*t*_R_ 18.8 min, 15 mg) and **3** (*t*_R_ 21.5 min, 15 mg). F06 (3.4 g) was subjected to silica gel CC (hexanes–acetone, 5:1 to 1:1, *v/v*) to give 16 subfractions (F06.01–F06.16). Subfraction F06.14 (20 mg) was purified by preparative HPLC (CHEMOSORB 5Si, CHCl_3_–MeOH, 9:1, *v/v*, 2 mL/min, UV 280, 300 nm) to afford compound **1** (*t*_R_ 30.7 min, 7 mg).

*9,10-Dihydro-5-methoxy-8-methyl-2,7-phenanthrenediol* (**1**). Light brown oil. UV (MeOH) λ_max_ (log ε) 298 (2.98), 279 (3.13) nm; ^1^H- and ^13^C-NMR data: see Table 1; HRESIMS *m/z* 257.1172 [M+H]^+^ (calcd. for C_16_H_17_O_3_^+^, 257.1172).

**Table 1:** ^1^H-NMR (400 MHz) and ^13^C-NMR (100MHz) data of compound **1** ^a^.

No.	δ_H_	δ_C_
1	6.62, d (2.4)	114.7
2	-	156.1
3	6.59, dd (8.6, 2.4)	113.6
4	7.95, d (8.6)	130.4
4a	-	126.7
4b	-	117.3
5	-	156.8
6	-	99.3
7	-	155.4
8	-	114.8
8a	-	140.40 ^b^
9	2.66, m	27.5
10	2.60, m	31.1
10a	-	140.43 ^b^
OCH_3_-5	3.78, s	56.1
CH_3_-8	2.11, s	11.6

^a^ in CD_3_OD, δ in ppm, *J* in Hz; ^b^ interchangeable.

*(2S,4′R,8′R)-3,4-*δ*-Dehydrotocopherol* (**2**). Brown oil. ${\left[\alpha \right]}_{D}^{25}$ +12 (*c* 0.1, EtOH); UV (MeOH) λ_max_ (log ε) 332 (4.0), 272 (4.2), 264 (4.3), 230 (4.8); CD (MeOH, *c* = 2.5 × 10^−3^ M) Δ*ε* (nm) +23.3 (278); ^1^H-NMR (CD_3_OD, 400 MHz) δ 6.46 (1H, d, *J* = 2.9 Hz, H-7), 6.32 (1H, d, *J* = 2.9 Hz, H-5), 6.24 (1H, d, *J* = 9.6 Hz, H-4), 5.59 (1H, d, *J* = 9.6 Hz, H-3), 4.26 (1H, s, OH), 2.13 (3H, s, C*H_3_*-8), 1.62 (2H, m, H-1'), 1.52 (1H, m, H-12'), 1.46 (1H, m, H_a_-2'), 1.43–1.32 (2H, m, H-4' and 8'), 1.36 (1H, m, H_b_-2'), 1.34 (3H, s, CH_3_-2), 1.32 (1H, m, H_a_-6'), 1.32–0.98 (8H, m, H-3', 5', 7', and 9'), 1.27 (2H, m, H-10'), 1.15 (1H, m, H_b_-6'), 1.14 (2H, m, H-11'), 0.86 (6H, d, *J* = 6.4 Hz, CH_3_-12' and H_3_-13'), 0.841 (3H, d, *J* = 6.4 Hz, CH_3_-8' interchangeable with CH_3_-4'), 0.830 (3H, d, *J* = 6.6 Hz, CH_3_-4', interchangeable with CH_3_-8'); ^13^C-NMR (CDCl_3_) δ 148.5 (C-6), 145.0 (C-8a), 131.1 (C-3), 126.4 (C-8), 122.7 (C-4), 121.5 (C-4a), 117.0 (C-7), 110.2 (C-5), 78.0 (C-2), 41.01 (C-1'), 39.4 (C-11'), 37.5 (C-3', interchangeable with C-5', C-7', or C-9'), 37.4 (C-5', interchangeable with C-3', C-7', or C-9'), 37.34 (C-7', interchangeable with C-3', C-5', or C-9'), 37.29 (C-9', interchangeable with C-3', C-5', or C-7'), 32.8 (C-8'), 32.7 (C-4'), 28.0 (C-12'), 25.8 (CH_3_-2), 24.8 (C-10'), 24.4 (C-6'), 22.7 (CH_3_-12'), 22.6 (C-13'), 21.3 (C-2'), 19.8 (CH_3_-8'), 19.7 (CH_3_-4'), 15.5 (CH_3_-8); HRESIMS *m/z* 401.3416 [M+H]^+^ (calcd. for C_27_H_45_O_2_, 401.3414).

*(2R,4′R,8′R)-3,4-*δ*-Dehydrotocopherol* (**3**). Brown oil. ${\left[\alpha \right]}_{D}^{25}$ −13 (*c* 0.1, EtOH); UV (MeOH) λ_max_ (log ε) 331 (3.9), 273 (4.1), 264 (4.2), 232 (4.8); CD (MeOH, *c* = 2.5 × 10^−3^ M) Δ*ε* (nm) −20.3 (275); ^1^H-NMR (CD_3_OD, 400 MHz) δ 6.47 (1H, d, *J* = 3.2 Hz, H-7), 6.32 (1H, d, *J* = 3.2 Hz, H-5), 6.24 (1H, d, *J* = 9.8 Hz, H-4), 5.58 (1H, d, *J* = 9.8 Hz, H-3), 4.27 (1H, s, OH), 2.13 (3H, s, CH_3_-8), 1.61 (2H, m, H-1'), 1.52 (1H, m, H-12'), 1.41 (2H, m, H_2_-2'), 1.42–1.32 (2H, m, H-4' and 8'), 1.34 (3H, s, CH_3_-2), 1.32 (1H, m, H_a_-6'), 1.32–0.98 (8H, m, H-3', 5', 7', and 9'), 1.26 (2H, m, H-10'), 1.16 (1H, m, H_b_-6'), 1.14 (2H, m, H-11'), 0.86 (6H, d, *J* = 6.4 Hz, CH_3_-12' and H_3_-13'), 0.834 (3H, d, *J* = 6.4 Hz, CH_3_-8' and CH_3_-4'); ^13^C-NMR (CDCl_3_) δ 148.5 (C-6), 145.0 (C-8a), 131.1 (C-3), 126.4 (C-8), 122.7 (C-4), 121.5 (C-4a), 117.0 (C-7), 110.2 (C-5), 78.0 (C-2), 40.97 (C-1'), 39.4 (C-11'), 37.4 (C-3', interchangeable with C-5', C-7', or C-9'), 37.4 (C-5', interchangeable with C-3', C-7', or C-9'), 37.34 (C-7', interchangeable with C-3', C-5', or C-9'), 37.35 (C-9', interchangeable with C-3', C-5', or C-7'), 32.8 (C-8'), 32.7 (C-4'), 28.0 (C-12'), 25.8 (CH_3_-2), 24.8 (C-10'), 24.5 (C-6'), 22.7 (CH_3_-12'), 22.6 (C-13'), 21.3 (C-2'), 19.8 (CH_3_-8'), 19.6 (CH_3_-4'), 15.5 (CH_3_-8); HRESIMS *m/z* 401.3412 [M+H]^+^ (calcd. for C_27_H_45_O_2_, 401.3414).

### 3.4. Cell Culture

The mouse fibroblasts NIH3T3 and HeLa human cervical cancer cells were obtained from the American Type Culture Collection (Manassas, VA, USA). The NIH3T3 cells displayed fibroblastic morphology and HeLa cells were polygonal. Both cells were tightly adherent to the flask, and were highly proliferative. NIH3T3 cells were cultured in Dulbecco’s Modified Eagle’s Medium (DMEM, Welgene, Gyeongsan, Korea) supplemented with 10% bovine calf serum (HyClone, Logan, UT, USA) and 1% penicillin-streptomycin (HyClone), HeLa cells were maintained in DMEM supplemented with 10% fetal bovine serum (HyClone) and 1% penicillin-streptomycin. Cells were maintained at 37 °C with 5% CO_2_ in a humidified atmosphere. The cells were subcultured when 80% confluence was reached according to the manufacturer’s recommendation. The morphology of the cells was examined under a microscope.

### 3.5. Cell Proliferation Assay

For proliferation studies, cell number count was conducted in monolayer culture in 12-well culture plates using a hemocytometer. NIH3T3 or HeLa cells were seeded at an initial density of 1 × 10^5^ cells per mL medium containing DMSO only, 10 μM of all isolates, the mixure of **2** and **3**, or δ-tocopherol. Cells were trypsinized and then viable cell numbers were counted in triplicates for each group every 24 h. The data was recorded as the average of three independent experiments. For the dose-dependency of cell proliferative effects of compounds, NIH3T3 cells were seeded at an initial density of 1 × 10^5^ cells per mL medium containing all isolates, the mixture of **2** and **3**, or δ-tocopherol (0.5 μM–10 μM; control DMSO only) and the effect on cell growth was determined after 4 days by counting cell numbers. Cell numbers were counted in triplicate for each group, and three independent experiments were performed.

### 3.6. Statistical Analysis

All results were expressed as mean ± s.d. Student’s *t*-test was used for comparisons involving two groups. *p* < 0.05 was considered to indicate a statistically significant difference.

## 4. Conclusions

In the present phytochemical study, a new compound, 9,10-dihydro-5-methoxy-8-methyl-2,7-phenanthrenediol (**1**) was isolated from the roots of *S. tuberosa* Lour. The structure of **1** was elucidated by the interpretation of its spectroscopic data. In addition, (2*S*,4'*R*,8'*R*)-3,4-δ-dehydrotocopherol (**2**) and (2*R*,4'*R*,8'*R*)-3,4-δ-dehydrotocopherol (**3**) were separated for the first time from the stereoisomeric mixtures of **2** and **3** by preparative HPLC using a chiral column. CD experiments were utilized to determine the absolute configurations at C-2 of **2** and **3**. When all isolates and the mixture of **2** and **3** were evaluated for their cell proliferative effects, the pure isomers **2** and **3** exhibited remarkable effects, suggesting the two compounds as powerful wound healing agent candidates.

## Supplementary Materials

Supplementary materials can be accessed at: http://www.mdpi.com/1420-3049/20/04/5965/s1.
