# Supplementary material for: A New 9,10-Dihydrophenanthrene and Cell Proliferative 3,4-δ-Dehydrotocopherols from Stemona tuberosa
Source: Molecules. 2015 Apr 3;20(4):5965–74. doi: 10.3390/molecules20045965 (PMC6272770; doi:10.3390/molecules20045965)
Supplement: Supplementary file 1 [file molecules-20-05965-s001.pdf]

Supplementary Materials

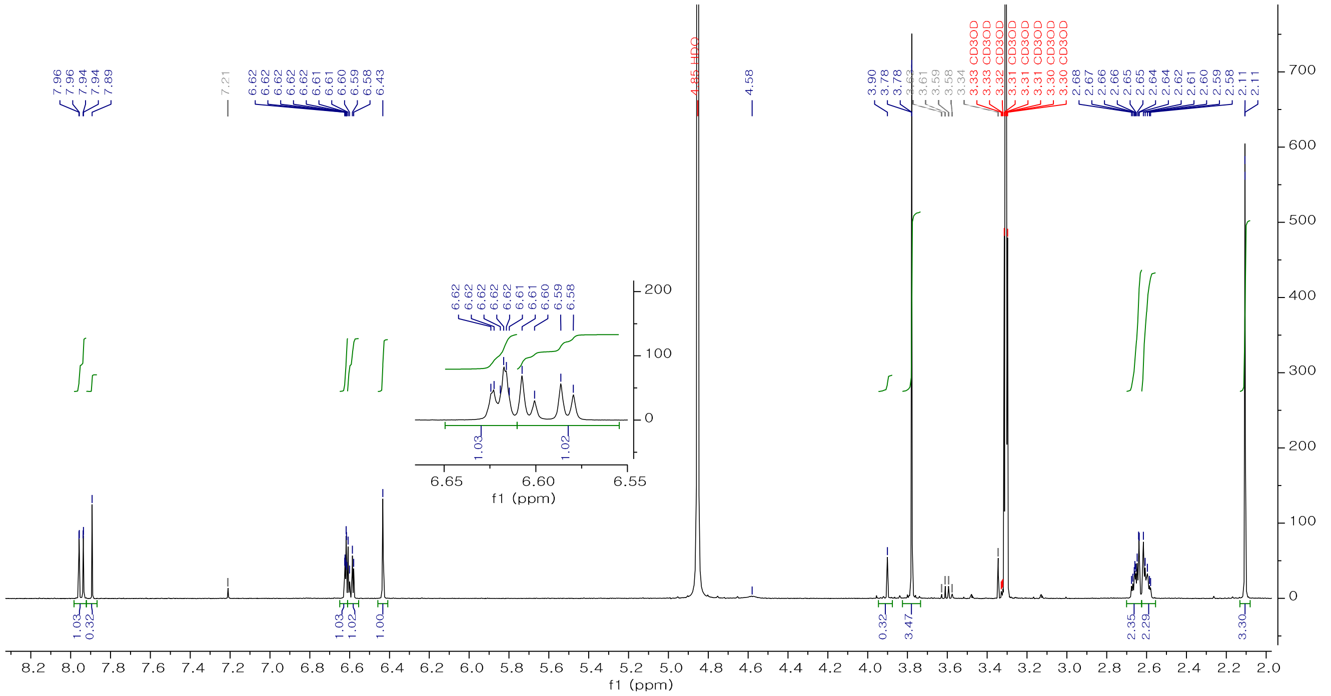

Figure S1. ^{1}H-NMR spectrum (400 MHz, CD_3OD) of **1**.

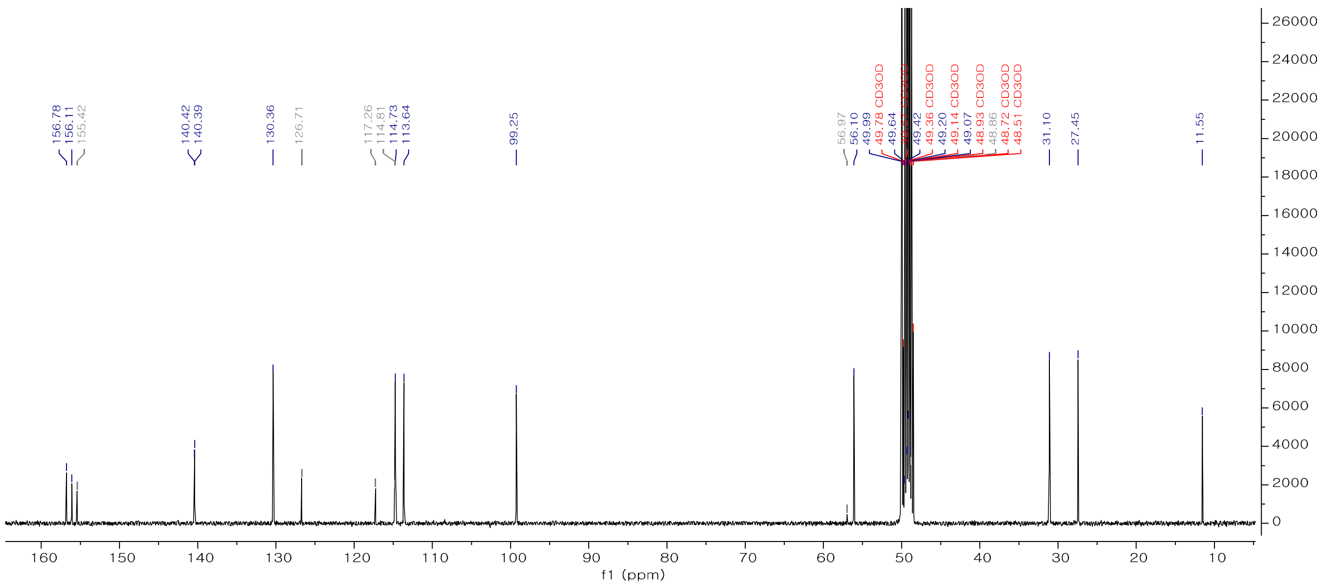

Figure S2. ^{13}C-NMR spectrum (100 MHz, CD_3OD) of **1**.

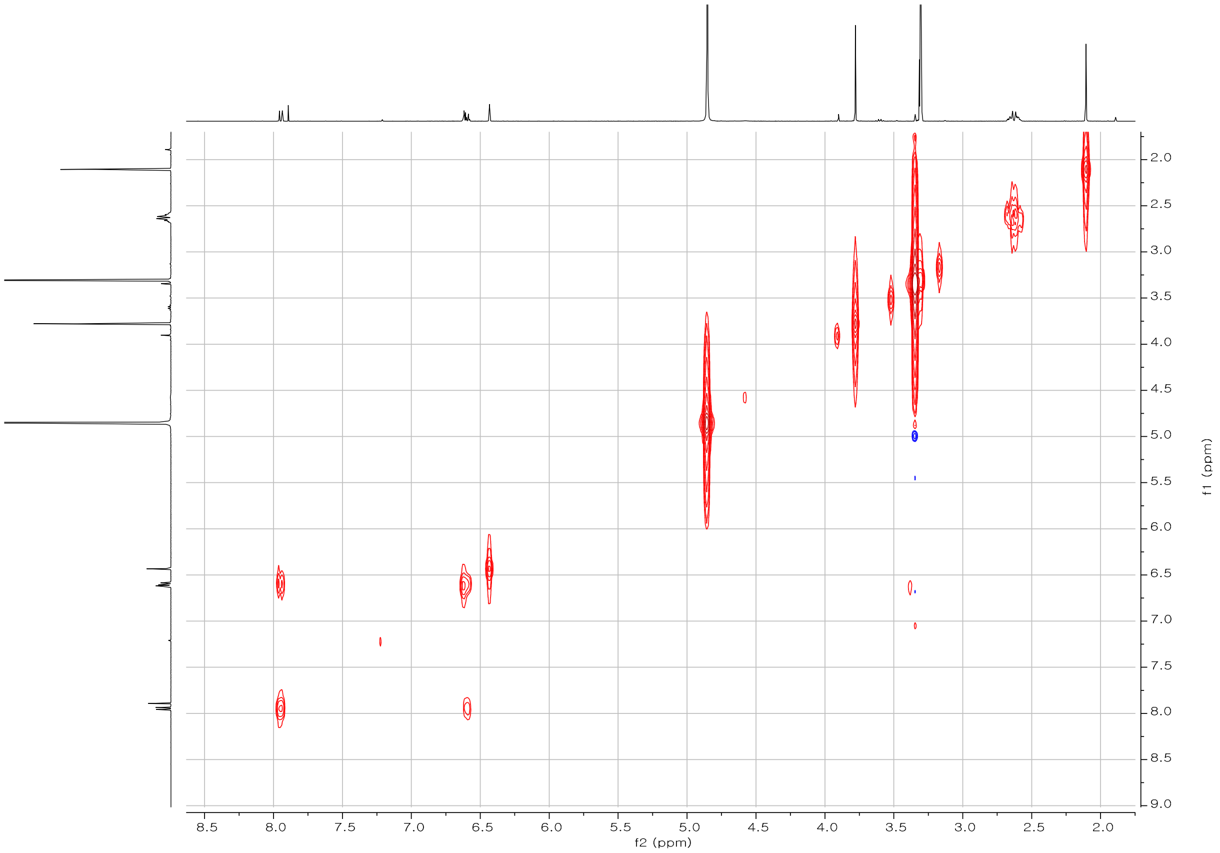

Figure S3. ^{1}H-^{1}H COSY NMR spectrum (CD_3OD) of **1**.

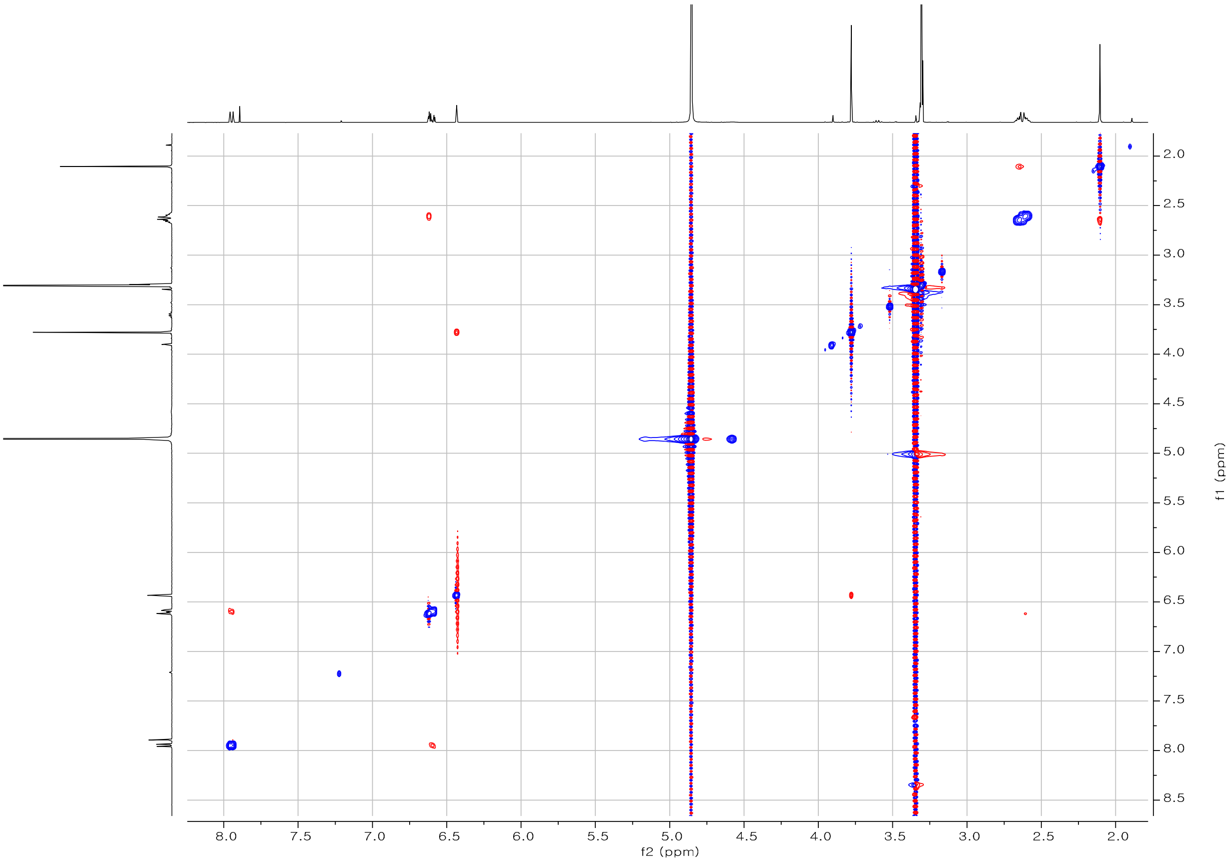

Figure S4. ^{1}H-^{1}H NOESY NMR spectrum (CD_3OD) of **1**.

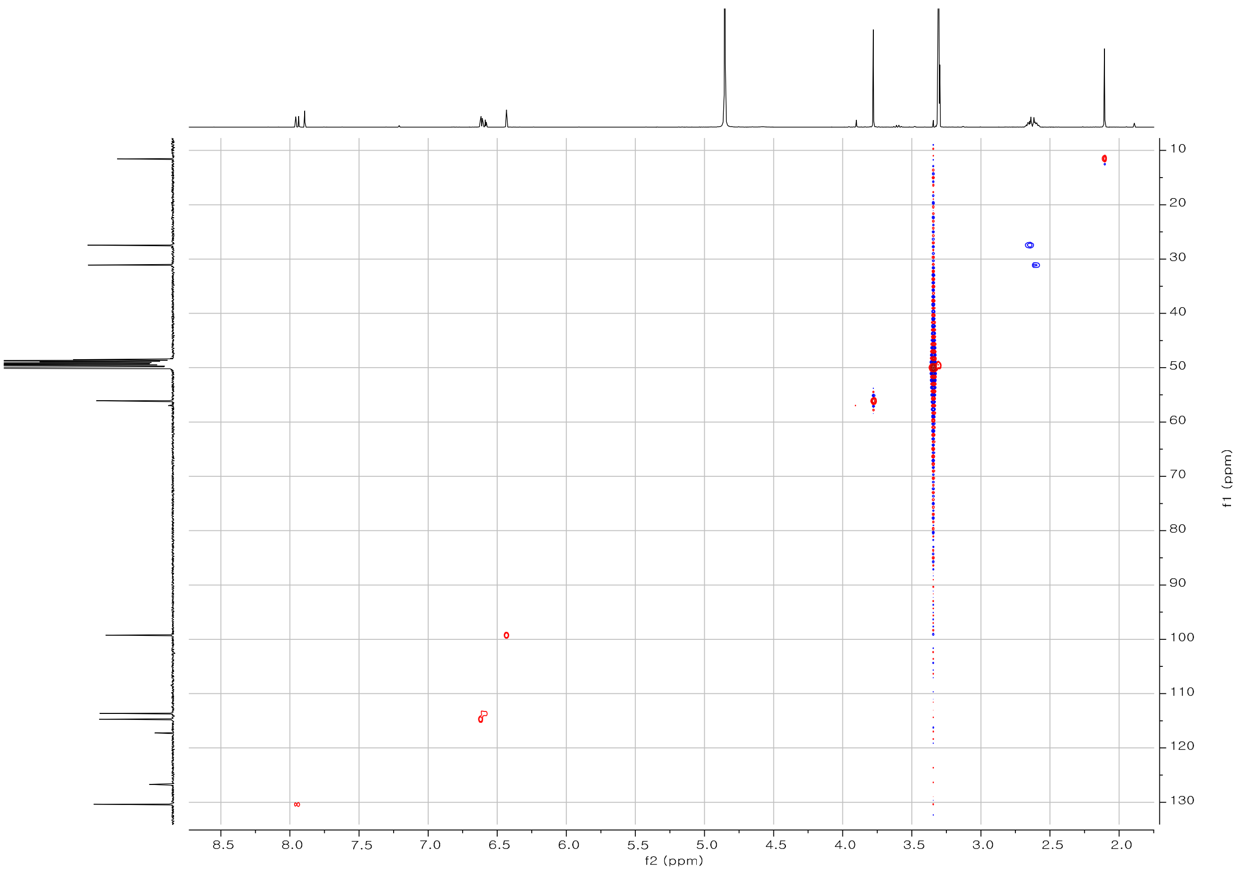

Figure S5. ^{1}H-^{13}C HSQC NMR spectrum (CD_3OD) of **1**.

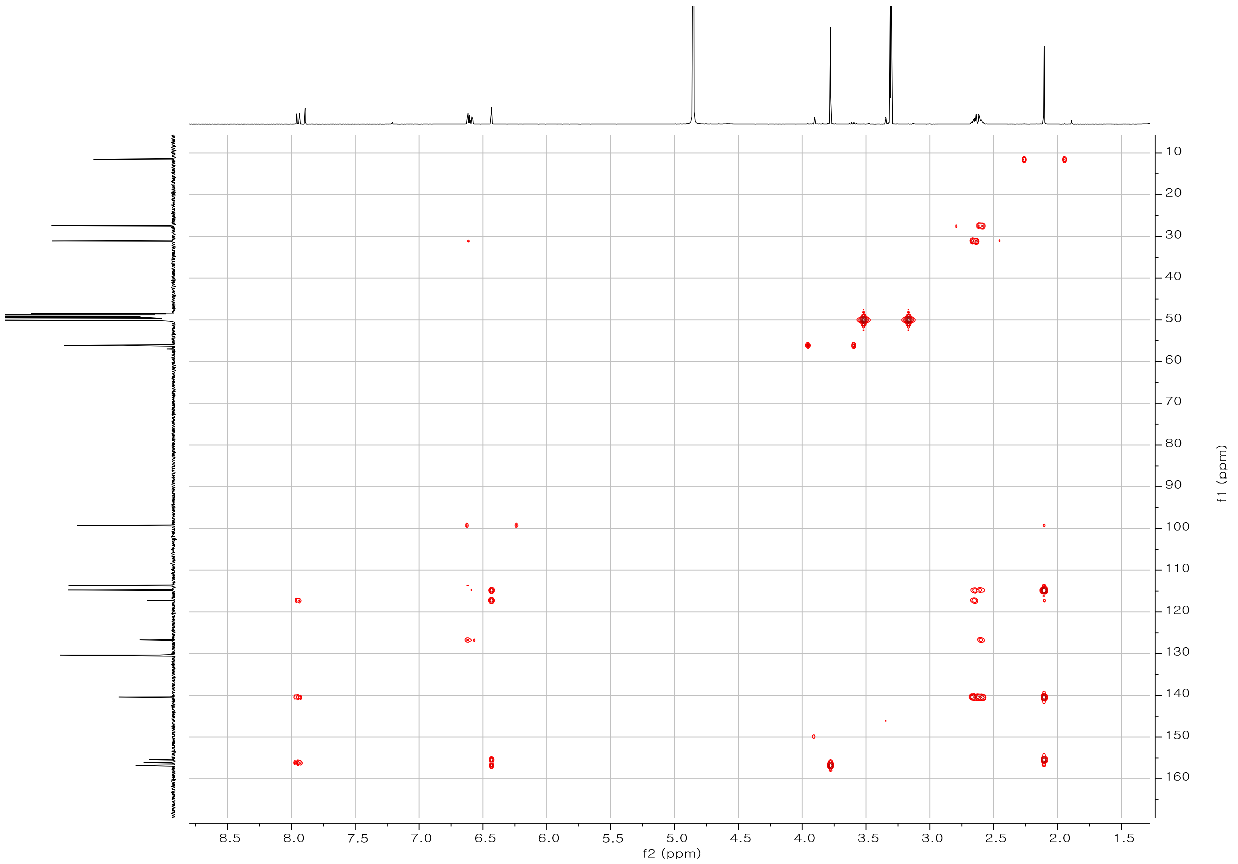

Figure S6. ^{1}H-^{13}C HMBC NMR spectrum (CD_3OD) of **1**.

Qualitative Compound Report

**Data File**	4(p)02.d	**Sample Name**	SampleName
**Sample Type**	Sample	**Position**	P1-E1
**Instrument Name**	Instrument 1	**User Name**	
**Acq Method**		**Acquired Time**	10/5/2012 11:22:42 AM
**IRM Calibration Status**	Success	**DA Method**	kikisil.m
**Comment**	vol. 0.01		

Compound Table

Compound Label	RT	Mass	Abund	Formula	Tgt Mass	Diff (ppm)
Cpd 1: C16H16O3	0.123	256.1099	4703	C16H16O3	256.1099	0.01

Compound Label	RT	Algorithm	Mass
Cpd 1: C16H16O3	0.123	Find By Formula	256.1099

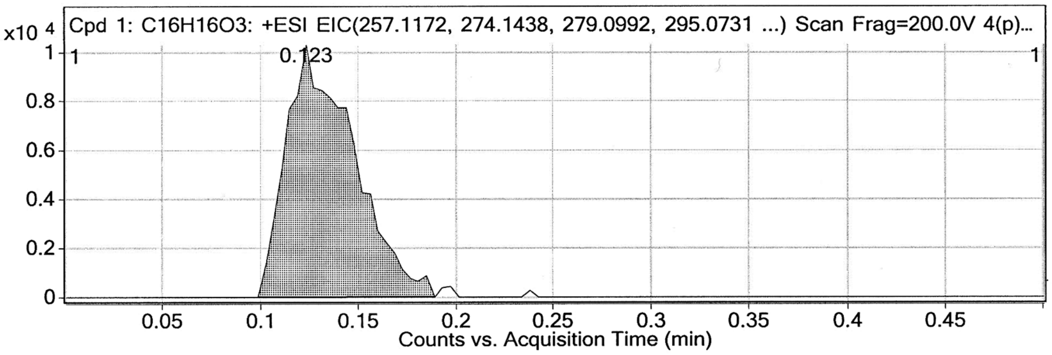

MS Spectrum

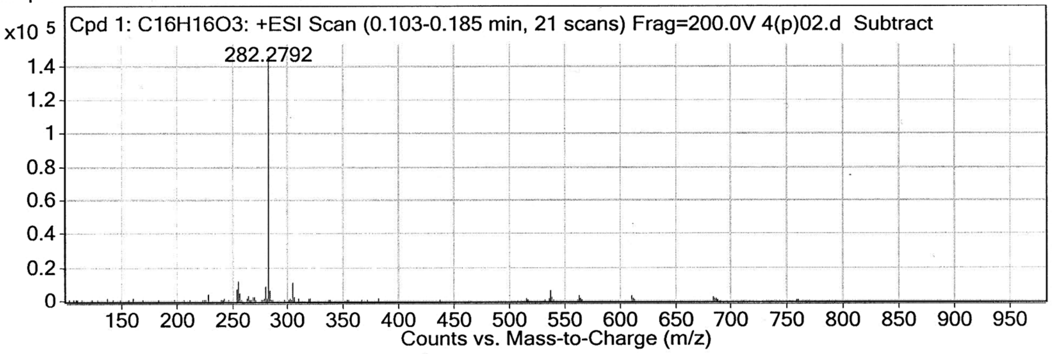

MS Zoomed Spectrum

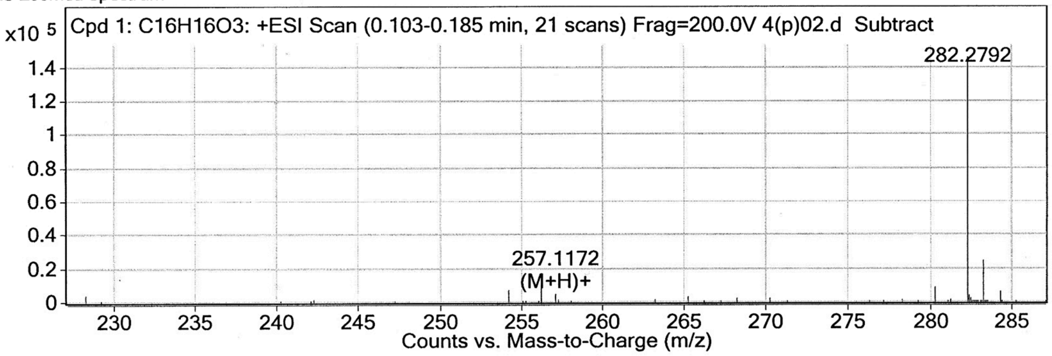

MS Spectrum Peak List

m/z	*Calc m/z*	Diff(ppm)	z	Abund	Formula	Ion
257.1172	257.1172	-0.02	1	4703	C16 H17 O3	(M+H)+

Figure S7. HRESIMS spectrum of **1.**
